# Flavonoid Rutin Increases Thyroid Iodide Uptake in Rats

**DOI:** 10.1371/journal.pone.0073908

**Published:** 2013-09-04

**Authors:** Carlos Frederico Lima Gonçalves, Maria Carolina de Souza dos Santos, Maria Gloria Ginabreda, Rodrigo Soares Fortunato, Denise Pires de Carvalho, Andrea Claudia Freitas Ferreira

**Affiliations:** Laboratório de Fisiologia Endócrina Doris Rosenthal, Instituto de Biofísica Carlos Chagas Filho, Universidade Federal do Rio de Janeiro, Rio de Janeiro, Brasil; University of Oslo, Norway

## Abstract

Thyroid iodide uptake through the sodium-iodide symporter (NIS) is not only an essential step for thyroid hormones biosynthesis, but also fundamental for the diagnosis and treatment of different thyroid diseases. However, part of patients with thyroid cancer is refractory to radioiodine therapy, due to reduced ability to uptake iodide, which greatly reduces the chances of survival. Therefore, compounds able to increase thyroid iodide uptake are of great interest. It has been shown that some flavonoids are able to increase iodide uptake and NIS expression *in vitro*, however, data *in vivo* are lacking. Flavonoids are polyhydroxyphenolic compounds, found in vegetables present in human diet, and have been shown not only to modulate NIS, but also thyroperoxidase (TPO), the key enzyme in thyroid hormones biosynthesis, besides having antiproliferative effect in thyroid cancer cell lines. Therefore, we aimed to evaluate the effect of some flavonoids on thyroid iodide uptake in Wistar rats *in vivo*. Among the flavonoids tested, rutin was the only one able to increase thyroid iodide uptake, so we decided to evaluate the effect of this flavonoid on some aspects of thyroid hormones synthesis and metabolism. Rutin led to a slight reduction of serum T4 and T3 without changes in serum thyrotropin (TSH), and significantly increased hypothalamic, pituitary and brown adipose tissue type 2 deiodinase and decreased liver type 1 deiodinase activities. Moreover, rutin treatment increased thyroid iodide uptake probably due to the increment of NIS expression, which might be secondary to increased response to TSH, since TSH receptor expression was increased. Thus, rutin might be useful as an adjuvant in radioiodine therapy, since this flavonoid increased thyroid iodide uptake without greatly affecting thyroid function.

## Introduction

The interest in new compounds with potential health properties has increased notably in the last decades. Flavonoid is a class of natural aromatic compounds produced by plants, which are found in our daily diet, such as nuts, grains and fruits. Flavonoids were shown to have important effects in mammals, including antioxidant, estrogenic/antiestrogenic, antiproliferative and have been shown to decrease the risk for stroke [1–3]. Although flavonoids could have beneficial effects, there are data suggesting that some of them could interfere with the thyroid axis [4–8].

Normal thyroid hormone synthesis requires iodide, which is transported into the thyrocyte through the sodium-iodide symporter (NIS), a glycoprotein expressed in the basolateral membrane of the cell. In the apical surface of the thyrocyte, iodide is oxidized and organified by the thyroperoxidase (TPO) enzyme. TPO requires hydrogen peroxide as co-substrate, which is generated by dual oxidase (DUOX) [9]. Previous data have shown that some flavonoids and extract of plants rich in flavonoids are able to inhibit TPO activity *in vitro* [10–12]. Moreover, it has been demonstrated that some flavonoids could affect thyroid hormone action and transport [13]. It has also been shown that some flavonoids can inhibit D1 activity [8,14,15], while flavonoids, such as fisetin, quercetin and kaempferol, stimulate D2 activity in RMS-13 cells [16]. Some flavonoids have also been shown to modulate NIS function and expression in cell culture models [17,18]. However, the *in vivo* effect of flavonoids on NIS function and expression has not been evaluated so far.

Rutin (5, 7, 3’, 4’-OH, 3-rutinose) is the glycosidic form of quercetin, being classified as a flavonol [19]. It is highly consumed [20], not only in food, but also due to its pharmacological properties, such as antitumor, anti-inflammatory, myocardial protective, antihypertensive, antiviral, antioxidant and even as an adjuvant for type 2 diabetes treatment [21–23]. Rutin has been used in the treatment of peripheral vascular diseases, because of its vascular-protective property [24]. Despite these beneficial properties, in 1996, Divi and Doerge [10] demonstrated that rutin is able to inhibit TPO iodination activity *in vitro*. Furthermore, our group showed that this flavonoid could also inhibit thyroid D1 activity *in vitro* [15]. However, to our knowledge, there is no data about the effect of this compound on thyroid function *in vivo*. Therefore, the aim of this work was to evaluate the effects of the *in vivo* treatment with the flavonoid rutin on serum thyroid hormones and TSH levels, thyroid TSH receptor expression, TPO and DUOX activity and the activity of the enzymes involved in peripheral thyroid hormone metabolism, D1 and D2. We also aimed to evaluate the *in vitro* effect of rutin on TPO iodide-oxidation activity kinetics. Moreover, since Na^+^/I^-^ symporter function is not only fundamental for thyroid hormone biosynthesis, but also a key element in the diagnosis and treatment of thyroid diseases, including thyroid cancer [25–27], we have also evaluated whether rutin could affect thyroid iodide uptake and NIS protein and mRNA levels.

## Methods

### Animals

Adult male Wistar rats were housed at controlled temperature (23±1°C) with daily exposure to a 12h light-dark cycle and free access to water and standard rat chow. This investigation conforms to the Guide for the Care and Use of Laboratory Animals published by the US National Institutes of Health (NIH Publication No. 85-23, revised 1996) and was approved by the Institutional Committee for Evaluation of Animal Use in Research (Comissão de Ética com o Uso de Animais (CEUA) em Experimentação Científica do Centro de Ciências da Saúde da Universidade Federal do Rio de Janeiro, number: IBCCF 135).

### Rutin Treatment

Three-months old male Wistar rats were divided into ten groups, with eight animals in each group, and treated with the following flavonoids: biochanin-A, catequin, fisetin, morin, naringenin, naringin, quercetin and rutin, all of them purchased from Sigma-Aldrich Co (St. Louis, MO, USA). Control groups received the vehicle used (propylene glycol). All flavonoids were dissolved in propylene glycol and daily administered, subcutaneously (sc), in the dose of 10mg/Kg body weight (BW) for five days [28]. Since rutin was the only flavonoid able to increase NIS function among the flavonoids tested, we have also evaluated whether a higher dose of rutin (20mg/Kg BW) would produce an additional increment in thyroid iodide uptake. Since we have found a greater effect using the higher dose, we then decided to evaluate the effect of daily injections of 20mg/Kg BW rutin, sc, for five days on thyroid function, thyroid hormone levels and metabolism, and serum TSH concentration.

### Thyroid iodide uptake

We have previously demonstrated that the measurement of radioiodide uptake 15 min after ^125^I–NaI administration (short-term iodide uptake) reflects iodide transport through the sodium-iodide symporter without the influence of *in vivo* thyroid iodine organification activity [29]. Thus, in order to evaluate the *in vivo* NIS function using thyroid radioiodine uptake measurements, the animals received Na-^125^I (3700 Bq i.p., Amersham, Buckinghamshire, England) 15 min before decapitation. Thyroids were removed and weighed. The radioactivity of the thyroid glands was measured using a gamma counter (LKB), and the percentage of the ^125^I in the gland relative to the total ^125^I injected was calculated.

### Thyroperoxidase activity

TPO extraction and activity measurement were performed as previously described [30–32]. Rat thyroids were minced and homogenized in 0.5 ml of 50 mM Tris-HCl buffer, pH 7.2, containing 1 mM KI, using an Ultra-Turrax homogenizer (Staufen, Germany). The homogenate was centrifuged at 100,000 g, 4^°^C for 1 h. The pellet was suspended in 0.5 ml triton (0.1% v/v) and incubated at 4^°^C for 24 h to solubilize TPO. The suspension was centrifuged at 100,000 g, 4^°^C for 1 h, and the supernatant containing solubilized TPO was used for the assays. Protein content was determined by the method of Bradford [33].

In order to measure TPO iodide-oxidation activity, the assay mixture contained: 1.0 ml of freshly prepared 50 mM sodium phosphate buffer, pH 7.4, 24 mM KI and 11 mM glucose, and increasing amounts of solubilized TPO. The final volume was adjusted to 2.0 ml with 50 mM sodium phosphate buffer, pH 7.4, and the reaction was started by the addition of 10 µl of 0.1% glucose oxidase (Boehringer Grade I). The increase in absorbance at 353 nm (tri-iodide production) was registered for 3 min on a Hitachi spectrophotometer (U-3300). The ΔA_353nm_/min was determined from the linear portion of the reaction curve and related to protein concentration. One unit of activity corresponds to ΔA_353nm_/min = 1.0.

### TPO iodide-oxidation activity inhibition in vitro

The TPO iodide-oxidation activity was measured as described above. The control assay mixture contained 1.0 ml of freshly prepared 50 mM sodium phosphate buffer, pH 7.4, containing 24 mM KI and 11 mM glucose, and the amount of solubilized TPO producing an iodide oxidation activity of 0.1 ΔA_353nm_/min. The final volume was adjusted to 2.0 ml with 50 mM sodium phosphate buffer, pH 7.4, and the reaction was started by the addition of 10 µl 0.1% glucose oxidase (Boehringer Grade I). The increase in absorbance at 353 nm (tri-iodide production) was registered for 4 min on a Hitachi spectrophotometer (U-3300). In order to test the inhibitory effects, the desired amounts of rutin solution were added to the assay mixture before adjusting the final volume to 2 ml. The ΔA_353nm_/min in the presence or absence of rutin was determined from the linear portion of the reaction curve.

The inhibitory activity was expressed as the concentration of rutin necessary to produce a 50% inhibition of the original peroxidase activity (IC_50_). The flavonoid was tested in at least three series of experiments, in which eight to 12 different concentrations were assayed.

### Iodide oxidation inhibitory kinetics

In order to evaluate the kinetic parameters of TPO-catalyzed iodide-oxidation inhibition, a given TPO activity was assayed as described above, with or without 3.4 µM rutin (IC_50_) and variable iodide concentrations. Each iodide concentration was tested three times in the presence or absence of rutin solution, and the ΔA_353nm_/min obtained were plotted against KI concentrations.

### DUOX preparation and activity

For dual oxidase (DuOx) preparation, the excised thyroid glands remained at 4°C for 24h in 50 mM sodium phosphate buffer, pH 7.2, containing 0.25 M sucrose, 0.5 mM DTT, 1 mM EGTA, 5 µg/ml aprotinin and 34.8 µg/ml PMSF before homogenization. Thyroids were homogenized in the same buffer, and then centrifuged at 100,000 g for 1h at 4°C. The pellet was suspended in 0.5 ml of 50 mM sodium phosphate buffer, pH 7.2, containing 0.25 M sucrose, 2 mM MgCl_2_, 5 µg/ml aprotinin and 34.8 µg/ml PMSF and stored at -70°C until H_2_O_2_ generation measurements [34].

H_2_O_2_ generation was quantified in thyroid particulate fractions by the Amplex red/horseradish peroxidase assay (Molecular Probes, Invitrogen), which detects the accumulation of a fluorescent oxidized product, as previously described [35]. Particulate fractions were incubated with 0.3 M sodium phosphate buffer, pH 7.2, containing 1 mM EGTA, 100 U/ml superoxide dismutase (Sigma), 0.5 U/ml horseradish peroxidase (Roche), 50 µM Amplex red, 1 mM NADPH, in the presence or absence 0.1 M CaCl_2_, and the fluorescence was registered on a spectrofluorimeter (Victor3, PerkinElmer). The excitation and emission wavelengths were 530 and 595nm, respectively. Specific DuOx activity was calculated by the difference between the activities in the presence and absence of calcium and was expressed per mg protein (nmoles H_2_O_2_.h^-1^.mg^-1^ protein).

### Rutin antioxidant effect

To study if 3.4 µM rutin (IC_50_ of TPO activity) would be able to scavenge hydrogen peroxide, 0.3 ?M H_2_O_2_ (Merck) was incubated with 100 U/ml superoxide dismutase (Sigma), 0.5 U/ml horseradish peroxidase (Roche), and 50 µM Amplex red, in the presence or absence of 3.4 µM rutin. The fluorescence was registered on a spectrofluorimeter (Victor3, PerkinElmer). The excitation and emission wavelengths were 530 and 595nm, respectively.

### Serum TSH

Serum TSH levels were evaluated by a specific RIA obtained from the National Institute of Diabetes, Digestive and Kidney Diseases (NIDDK – Bethesda, USA), and expressed in terms of the reference preparation 2 (RP-2). Intra- and interassay coefficients of variation were 7.7 and 6.5%, respectively, and the sensitivity was 0.63 ng/ml.

### Serum total T3

Serum total T3 concentrations were measured using commercial kit, based on the presence of specific antibodies adhered to the internal surface of propylene tubes (COAT-A-COUNT^®^ Total T_3_, Los Angeles, EUA). Sensitivity was 0.1nmol/L and inter- and intra assay coefficients of variation varied from 8.3 to 8.6% e 2.9 to 3.3%, respectively. All the procedures were carried out following the recommendations of the kits.

### Serum total and free T4

Serum total and free T4 concentrations were measured by electrochemical Luminescence commercial kit (FT4 and T4, Roche Diagnostics GmbH, Manheim, Germany). Sensitivity varied from 0.023 to 7.77ng/dL and 0.420 to 24.86 µg/dL for FT4 and T4 respectively. The inter- and intra assay coefficients of variation varied from 12.2 to 7.6% and 10.9 to 4.9%, respectively (according the biologic variation table Westgard). All the procedures were carried out following the recommendations of the kits.

### Type 1 and type 2 deiodinase activity

Liver, brown adipose tissue (BAT), pituitary and hypothalamus 5’-deiodinase activities were evaluated as previously described [36,37]. In short, 15 mg of tissue samples (liver or BAT), or the whole pituitary and hypothalamus were homogenized in 1 ml 0.1 M sodium phosphate buffer containing 1 mM EDTA (Merck), 0.25 M sucrose (Merck) and 10 mM dithiothreitol (DTT) (Sigma, USA), pH 6.9. For the measurement of type 1 deiodinase activity, homogenates (30 µg of protein from liver) were incubated in duplicate for 1 hour at 37°C (water bath) with 1 µM rT3 (Sigma, USA), equal volumes of [^125^I] rT3 (PerkinElmer Life Sciences, Boston, MA), previously purified using sephadex LH-20, and 10 mM DTT (Sigma, USA) in 100 mM potassium phosphate buffer containing 1 mM EDTA, pH 6.9, in a reaction volume of 300 µl. For type 2 deiodinase activity assay, homogenates (30 µg of protein from hypothalamus and pituitary and 20µg of protein from BAT) were incubated in duplicate for 3 hours at 37°C (water bath) with 1 nM T4 (Sigma, USA), and equal volumes of [^125^I] T4 (PerkinElmer Life Sciences, Boston, MA) previously purified using sephadex LH-20, in the presence of 1 mM PTU and 20 mM DTT (Sigma, USA) in 100 mM potassium phosphate buffer containing 1 mM EDTA, pH 6.9, in a reaction volume of 300 µl. In both cases, blank incubations were carried out in the absence of protein. After incubation, the reaction was stopped at 4°C, followed by the addition of 100 µl fetal bovine serum (Cultilab, BR) and 200 µl trichloroacetic acid (50% v/v). The samples were centrifuged at 8,000 g for 3 min and the supernatant was collected for measurement of ^125^I liberated during the deiodination reaction. Protein concentration in the samples was measured by the Bradford method [33], after incubation of homogenates with 2.5N NaOH.

### Immunoblotting for NIS and TSH receptor

Thyroids were homogenized in lysis buffer containing 135 mM NaCl, 1 mM MgCl_2_, 2.7 mM KCl, 20 mM Tris, pH 8.0, 1% triton, 10% glycerol, and protease and phosphatase inhibitors (0.5 mM Na _3_VO_4_, 10 mM NaF, 1 mM leupeptin, 1 mM pepstatin, 1 mM okadaic acid, and 0.2 mM phenylmethylsulfonyl fluoride), and then homogenized using an Ultra-Turrax homogenizer (Staufen, Germany). Subsequently, the samples were centrifuged at 570 g, 10 min, 4^°^C, and the supernatant was collected. An aliquot was used to determine the concentration of protein by the BCA protein assay kit (Pierce, Rockford, IL, USA, catalog number 23227), following the recommendations of the manufacturer. Protein samples (30 µg) were then resolved on SDS/PAGE electrophoresis, transferred to PVDF membranes, and probed with the indicated antibodies. NIS antibody (kindly provided by Dr. Nancy Carrasco) was diluted 1:2000, while the TSH receptor antibody (purchased from Santa Cruz Biotechnology - California, USA) was diluted at 1:500. GAPDH antibody (purchased from Millipore Corporation, California, USA) was used as internal control at dilution 1:5000. The second antibodies were utilized according manufactures instructions, anti-rabbit IgG HRP-linked antibody for NIS and anti-mouse IgG HRP-linked antibody (both secondary antibodies were purchased from Cell Signaling Technology) for TSH receptor and GAPDH. The immunoblots were revealed using ECL.

### Real-Time-PCR

Thyroid total RNA was extracted using the RNeasy® Plus Mini Kit (Qiagen), following the manufacturer’s instructions. After DNase treatment, reverse transcription was followed by real-time-PCR, as previously described [38]. Specific oligonucleotides, as described in Table 1, were purchased from Applied Biosystems (Foster City, California, USA). GAPDH was used as internal control.

**Table 1:** *Forward* and *Reverse* sequences for specific oligonucleotide genes.

**Genes**	***Forward***	***Reverse***
**GAPDH**	5’ – TGA TTC TAC CCA CGG CAA GT -3’	5’ – AGC ATC ACC CCA TTT GAT GT -3’
**NIS**	5’ – GCC CCA AAG GAA GAC ACT G -3’	5’ – CAT CGT GCC CCA GAT ACA G -3’
**TPO**	5’ – GAA TGA GGA ACT GAC CGA GAG -3’	5’ – TGA CAA GCC ACA GAA CTC TC -3’
**DUOX1**	5’ – ATT TCT TGG GAG GTA CAG CG -3’	5’ – GTT AGG CAG GTA GGG TTC TTT C -3’
**DUOX2**	5’ – TGC TCT CAA CCC CAA AGT G -3’	5’ – TCT CAA ACC AGT AGC GAT CAC -3’
**TSHR**	5’ – AGG TCC CTT GGA AAA ATG AGG -3’	5’ – GTC TCG AGT AGC TTC AGA GTC -3’

**GAPDH** (glyceraldehyde 3-phosphate dehydrogenase), **NIS** (sodium-iodide symporter), **TPO** (thyroperoxidase), **DUOX1** (Dual Oxidase 1), **DUOX2** (Dual Oxidase 2), and **TSHR** (thyrotropin receptor)

### Statistical analysis

The experiments were repeated three times, using at least 3 animals per group in each experiment, thus a total number of at least 9 animals per group were achieved. Results are expressed as mean ± SEM. Results of iodide uptake after treatment with different flavonoids were analyzed by the non-parametric Kruskal-Wallis test followed by the Dunn’s multiple comparison test, and the remainder data were analyzed by unpaired *t*-test. Statistical analyses were performed using the GraphPad Prism software (version 4, GraphPad Software, inc., San Diego, USA). Differences were considered significant when p<0.05.

## Results

### Effect of flavonoids on NIS function

**Figure 1:**
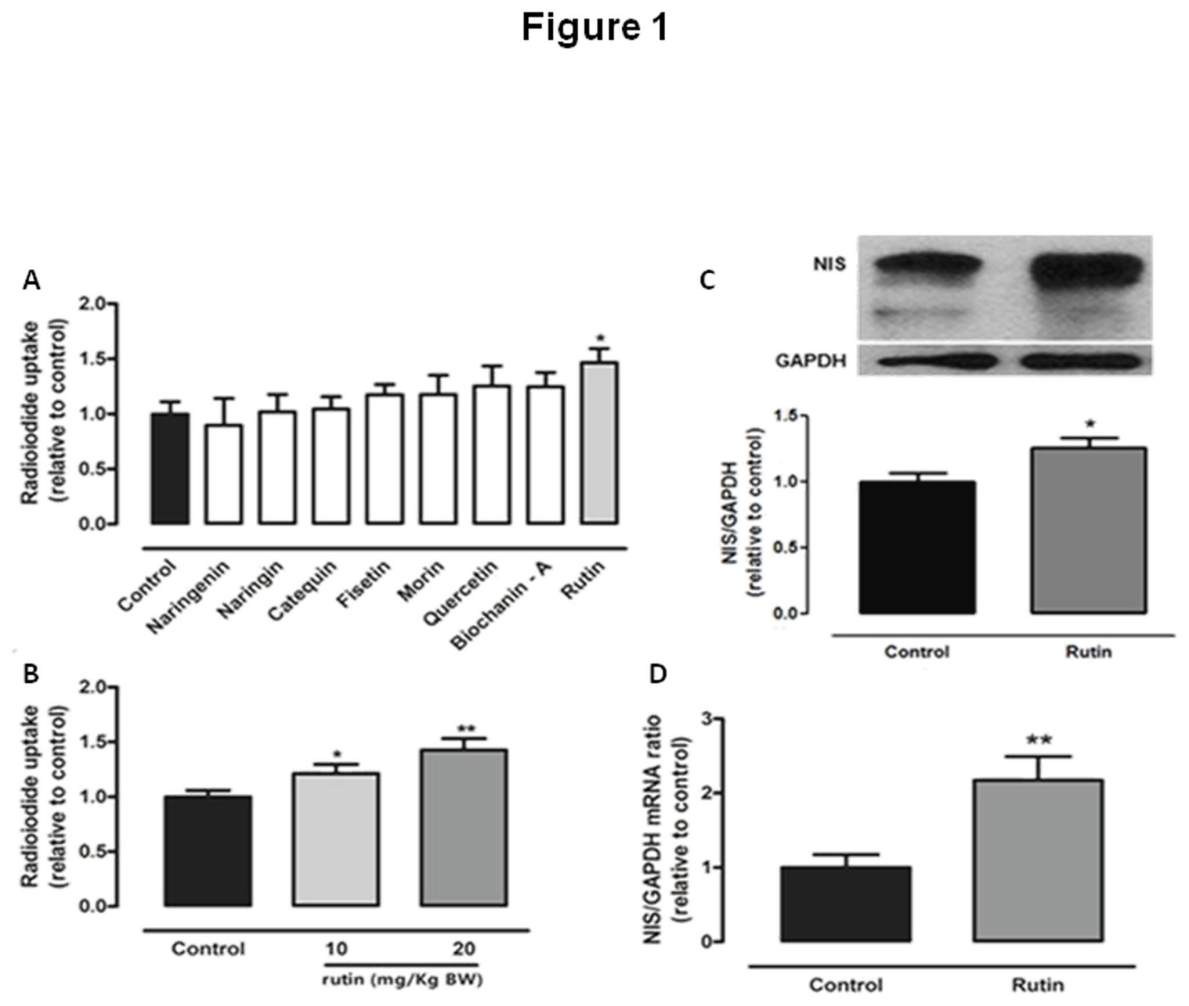
Effect of flavonoids on thyroid radioiodide uptake and effect of rutin on NIS. (A) Thyroid radioiodide uptake of rats treated with different flavonoids for five days (biochanin-A, catequin, fisetin, morin, naringenin, naringin, quercetin and rutin, 10mg/Kg body weight (BW), daily injections, sc.). Results are expressed as percentage of control group (n = 10 per group; *p < 0.05 vs. control); (B) Thyroid radioiodide uptake in rats treated with two different doses of rutin (10 or 20mg/Kg BW, daily injections, sc.) for five days. Results are expressed as percentage of control group (fold increase: rutin 10mg/Kg = 1.22; 20mg/Kg = 1.45; n = 15 per group; *p < 0.05 vs. control; **p < 0.01 vs. control); (C) Thyroid sodium-iodide symporter (NIS) protein levels in rats treated with 20mg/Kg BW rutin (daily injections, sc. for five days) (fold increase=1.34; n = 9 per group, *p < 0.05 vs. control); and (D) Thyroid NIS mRNA levels in rats treated with 20mg/Kg BW rutin (daily injections, sc. for five days) (fold increase=2.12; n = 10 per group, **p < 0.01 vs. control). GAPDH was used as internal control.

Since thyroid iodide uptake is a fundamental step for thyroid hormone synthesis, we have initially evaluated the effect of *in vivo* treatment with 10mg/Kg BW of some flavonoids (biochanin-A, catequin, fisetin, morin, naringenin, naringin, quercetin and rutin) on thyroid radioiodide uptake. We have observed that, among the flavonoids tested, only rutin was able to alter thyroid iodide uptake, increasing NIS function (Figure 1A).

### Effect of rutin on proteins involved in thyroid function

Subsequently, we have evaluated if the effect of rutin on NIS function is dose-dependent. So, we have treated rats with rutin in the doses of 10 and 20mg/Kg BW. As shown in Figure 1B, the *in vivo* treatment with rutin has improved thyroid iodide uptake in a dose-dependent manner. Thus, we decided to study the impact of 20mg/Kg BW rutin on thyroid function *in vivo*.

Rutin treatment increased not only thyroid iodide uptake, but also NIS protein (Figure 1C) and mRNA (Figure 1D) levels, suggesting that rutin was able to stimulate NIS expression.

**Figure 2:**
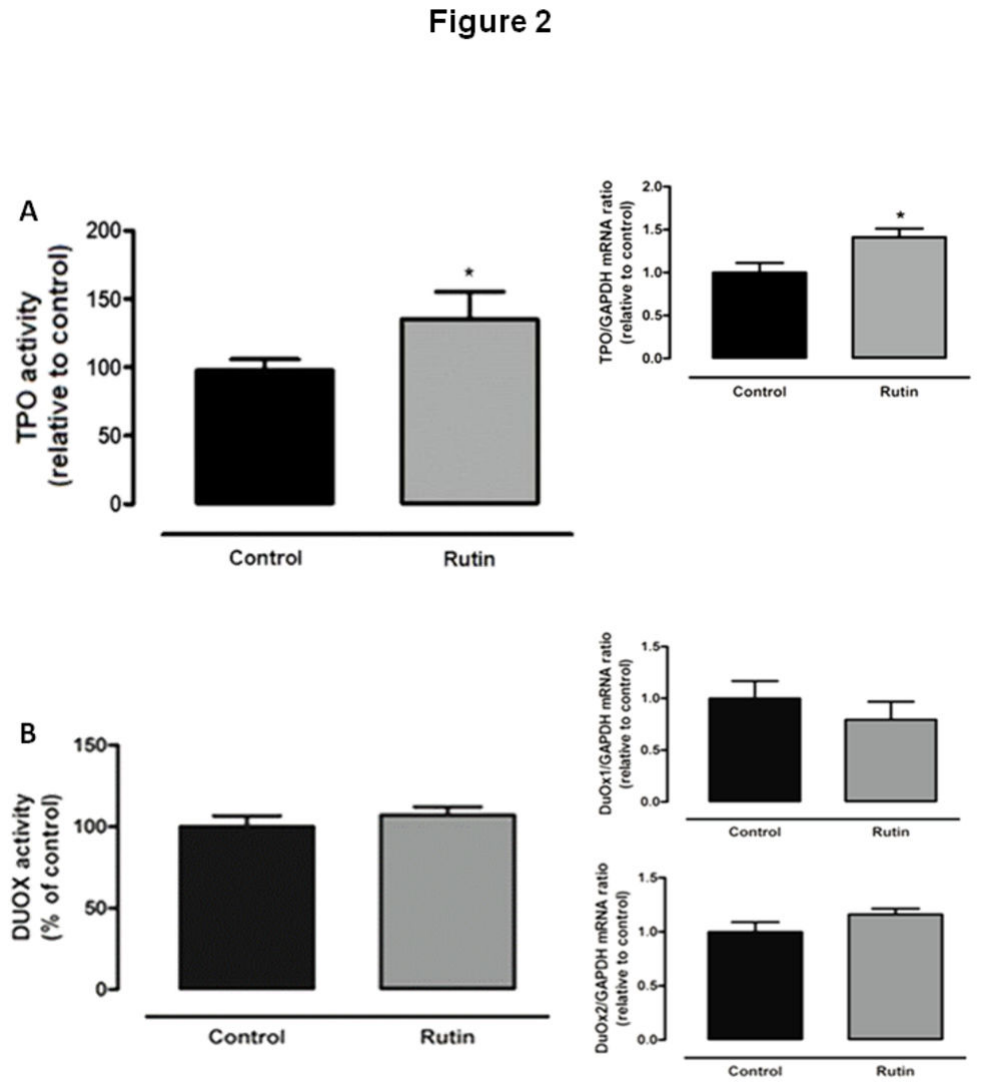
Effect of *in vivo* treatment with rutin on thyroid TPO and DUOX activity and mRNA. (A) Thyroperoxidase (TPO) activity (fold increase=1.35; n = 15 per group) and mRNA levels (fold increase=1.47; n = 10 per group) and (B) Thyroid Ca^++^ and NADPH -dependent H_2_O_2_ generation (n = 15 per group) and dual oxidase 1 (DUOX1) and 2 (DUOX2) mRNA levels (n = 10 per group) in rats treated with rutin (20mg/Kg BW, daily injections, sc.) for five days. DUOX activity and mRNA levels remained unchanged. *p < 0.05 vs. control.

Treatment with 20mg/Kg BW rutin for 5 days was able to increase *in vitro* TPO activity and the mRNA levels of thyroid TPO (Figure 2A), even though thyroid DUOX activity and DUOX1 and DUOX2 mRNA levels were not affected by rutin treatment (Figure 2B).

**Figure 3:**
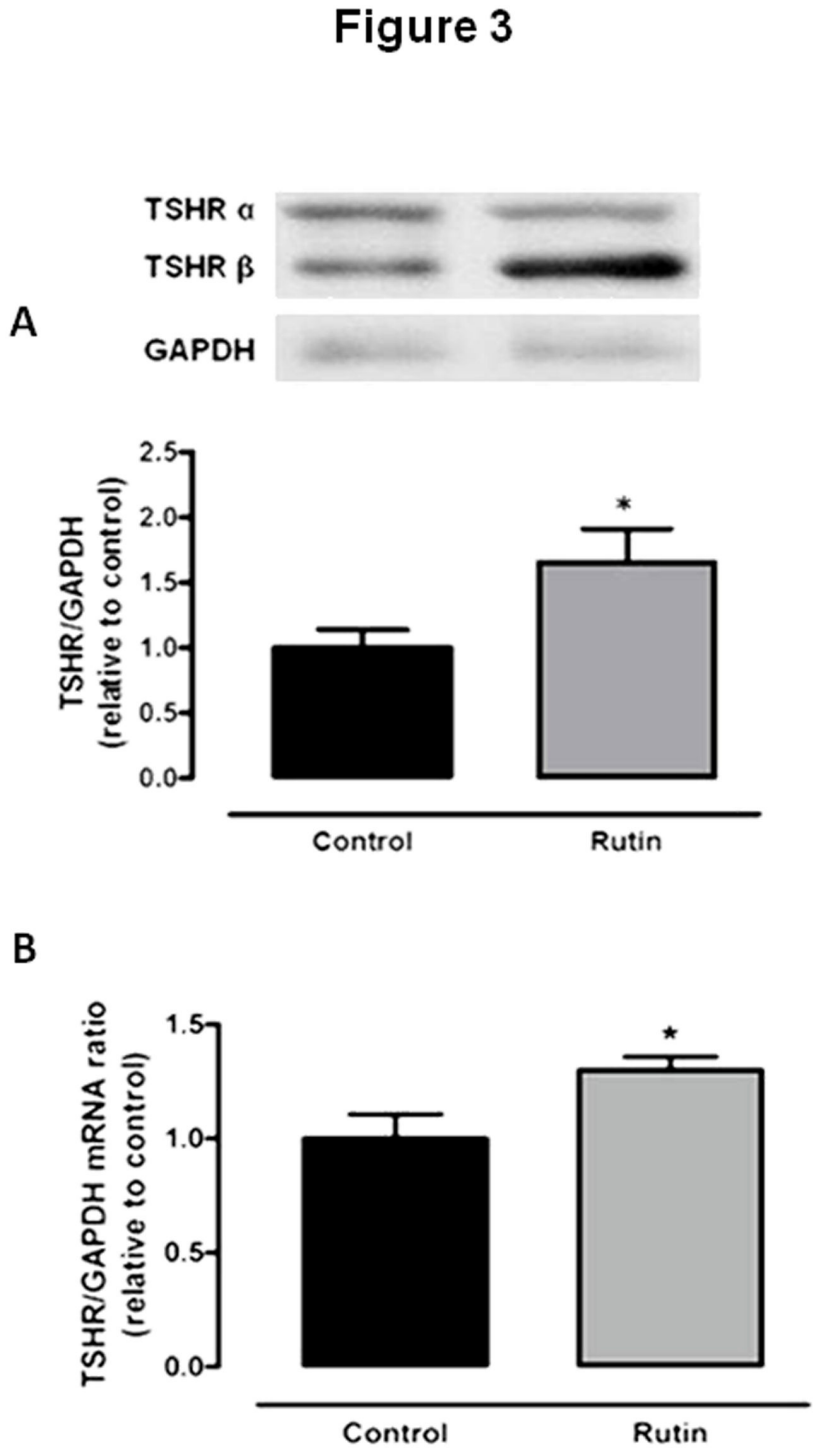
Effect of *in vivo* treatment with rutin on thyroid TSH receptor levels. (A) Thyroid TSH receptor (TSHR) protein (n = 12 per group) (B) and mRNA (fold increase=1.93; n = 10 per group) levels in rats treated with rutin (20mg/Kg BW, daily injections, sc.) for five days. GAPDH was used as internal control. *p < 0.05 vs. control.

TSHR protein (Figure 3A) and mRNA (Figure 3B) levels were increased in rats treated with rutin. Thus, rutin seems to up-regulate proteins important for thyroid hormone synthesis, NIS and TPO, probably because of increased sensitivity to TSH.

### Effect of rutin on serum thyroid hormones and TSH concentration

Even though rutin seems to stimulate thyroid function, surprisingly the serum total T4 and T3 concentrations were significantly decreased in rats treated with rutin, as shown in Table 2. Despite the decreased thyroid hormone levels, serum TSH level remained unchanged (Table 2). Previous data have shown that the synthetic flavonoid, EMD 21388, is able to compete with thyroid hormones for the binding to transthyretin (TTR), which is a protein important for thyroid hormones transport [39,40]. If rutin exerts the same effect, this could explain why TSH levels remained unchanged despite the decreased serum total thyroid hormone levels. Thus, we have decided to evaluate serum free T4 levels, which were also reduced in the serum of rutin-treated rats (Table 2), so the abnormally unaltered serum levels of TSH could not be explained by serum free T4 levels.

**Table 2:** Effect of rutin treatment on serum thyroid hormones and TSH concentrations.

**Group**	**Total T3 (ng/dl)**	**Total T4 (μg/dl)**	**Free T4 (μg/dl)**	**TSH (ng/dl)**
**Control**	44.80 ± 2.56	5.37 ± 0.11	2.90 ± 0.07	1.49 ± 0.16
**Rutin**	35.07 ± 1.96**	4.39 ± 0.12***	2.59 ± 0.08**	1.65 ± 0.26

Rats were treated with rutin (20mg/Kg BW, sc, 5 days) or vehicle (control group).

Results are expressed as mean±SEM. **p < 0.01 vs. control; ***p < 0.001 vs. control (n = 15 per group).

### Effect of rutin on in vitro thyroperoxidase activity

**Figure 4:**
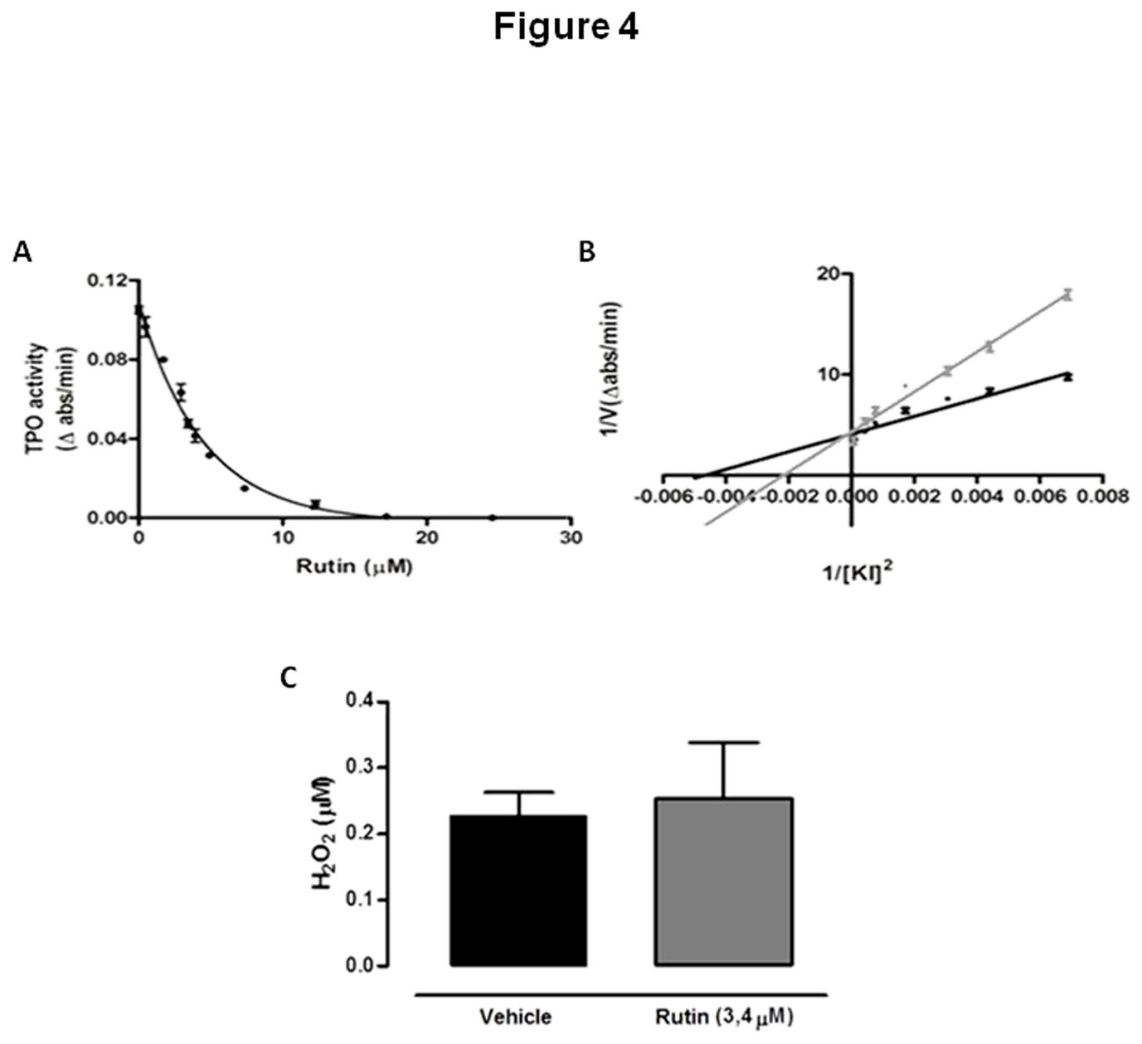
*In vitro* effect of rutin on TPO activity and on H_2_O_2_ added to medium. (A) Thyroperoxidase (TPO) iodide-oxidation activity was measured in the presence of different rutin concentrations; (B) The amount of solubilized TPO producing a fixed iodide oxidation activity (Δ_A353nm_/min=0.1) was assayed in the presence (grey) or absence (black) of 3.4 µM rutin (IC50). Different concentrations of KI were added, the final volume was adjusted to 2.0 ml and TPO iodide-oxidation activity was measured (V_máx_: rutin = 0.3052 ± 0.03240; control = 0.3129 ± 0.02702; K_(m)_: rutin = 44.93 ± 2.145; control = 26.52 ± 0.7015). (C) H_2_O_2_ concentration was measured after incubation with or without 3.4µM rutin (TPO IC50). H_2_O_2_ levels remained unchanged. All experiments were repeated at least three times.

Since serum thyroid hormone levels were reduced in rats treated with rutin, despite the increased thyroid iodide uptake, NIS expression and TPO activity and mRNA levels, we have also analyzed the *in vitro* effect of rutin on TPO iodide-oxidation activity. As shown in Figure 4A, TPO activity was significantly inhibited by rutin *in vitro*, as previously described [10] with a 50% inhibition of the original TPO activity (IC_50_) obtained at a 3.4 µM rutin concentration. This result suggests that the reduction of thyroid hormones in the serum of rats treated with rutin might be due, at least in part, to the *in vivo* decreased TPO activity.

The possibility of a TPO inhibition caused by competition with the substrate (iodide) was also evaluated. Kinetic iodide-oxidation studies showed that in the presence of 3.4 µM rutin TPO K_0.5_ for iodide was significantly increased (without rutin: 26.52 ± 0.70 mM; with rutin: 44.93 ± 2.14 mM, p < 0.05), but not V_max_ (without rutin: 0.31 ± 0.027 ?A_353_/min; with rutin: 0.30 ± 0.032 ? A_353_/min) (Figure 4B). Thus, rutin seems to reversibly inhibit TPO activity, by competition with the substrate, which could explain why the TPO activity was even higher when extracted from the thyroid of rats treated with rutin. Probably, during the processing of the gland, rutin is removed and the enzyme can resume its *in vitro* activity. Methimazole, which is largely used to treat hyperthyroidism due to its TPO-inhibitory effect, has been shown to reversibly inhibit TPO activity, so that *in vitro* TPO activity obtained from the thyroid of rats treated with methimazole has been shown to be normal [29] or increased in cultured porcine follicles treated with methimazole [41].

Since rutin has antioxidant properties [42,43], we then speculated if the inhibitory effect of rutin on TPO activity could also be due to decreased H_2_O_2_ availability. So, we have also tested if this flavonoid, in the concentration of 3.4 µM, would be able to scavenge H_2_O_2_. As shown in Figure 4C, at this concentration rutin was not able to significantly scavenge H_2_O_2_.

### Effect of rutin on type 1 and type 2 iodothyronine deiodinase activities

**Figure 5:**
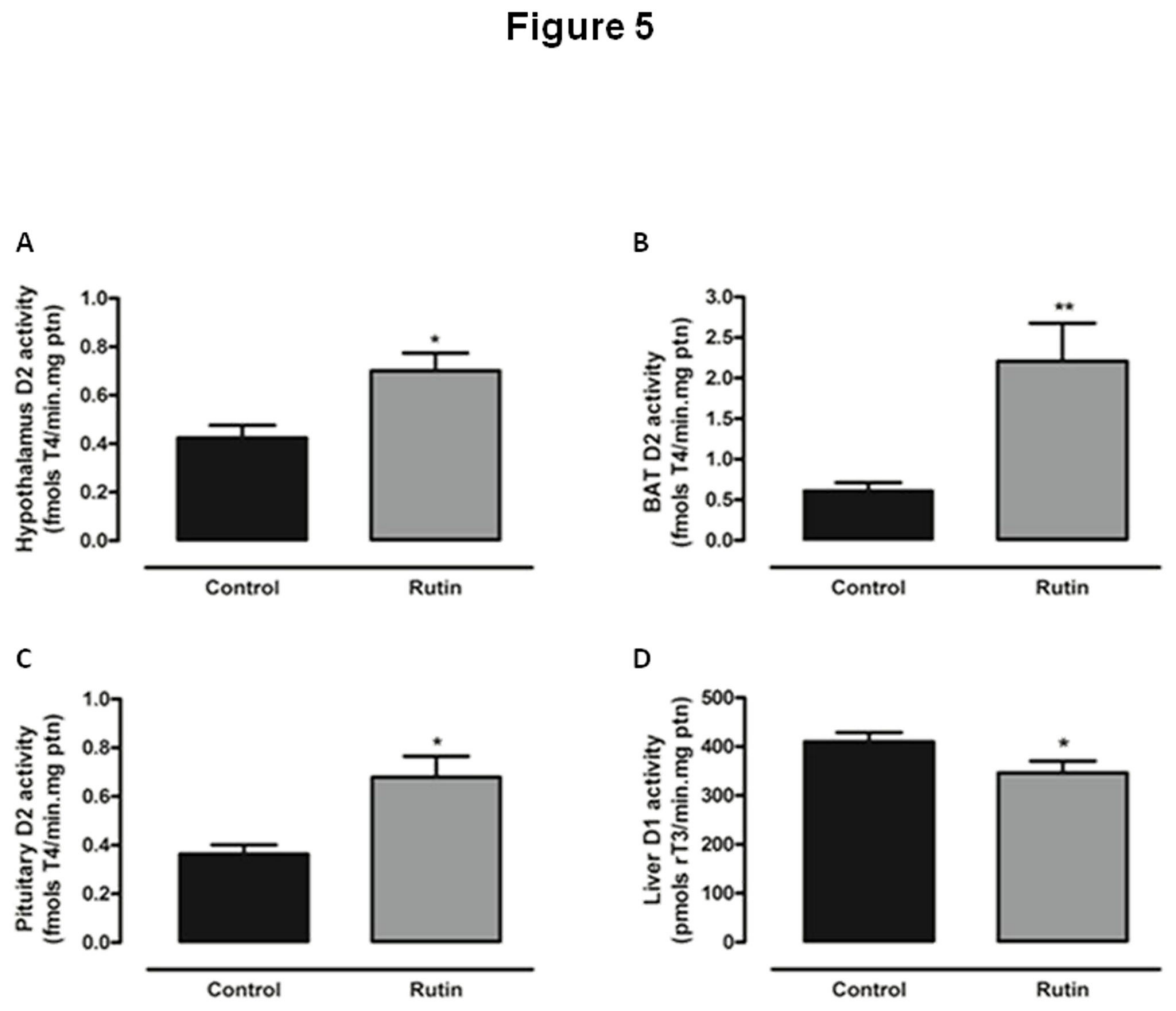
Effect of *in vivo* treatment with rutin on type 1 and type 2 deiodinase activity. 5’-deiodinase activity of (A) hypothalamic type 2 deiodinase (fold increase=1.65); (B) brown adipose tissue (fold increase=3.57), (C) pituitary D2 (fold increase=1.87) and (D) hepatic type 1 deiodinase (D1) of rats treated with rutin (20mg/Kg BW, daily injections, sc.) for five days (reduction of 29% related to control) (n = 15 per group for all assays, *p < 0.05 vs. control; ** p < 0.01 vs. control).

Serum thyroid hormones levels are determined by both synthesis and metabolism. Since type 1 and 2 iodothyronine deiodinases exert a fundamental role on peripheral metabolism of thyroid hormones, we have evaluated the effect of rutin treatment on liver D1 and on hypothalamic, pituitary and brown adipose tissue D2 activities. As shown in Figure 5A, B and C, rats treated with rutin showed significantly increased D2 activities in hypothalamus, pituitary and BAT, respectively. Moreover, a significant decrease in liver D1 activity by treatment with rutin was observed, as shown in Figure 5D. Liver D1 can be decreased either by a direct action of rutin, or secondary to decreased serum T3 levels. It is tempting to speculate whether serum TSH is maintained in the normal range due to a higher intra pituitary T3 generation secondary to the increased D2 activity.

## Discussion

Herein we have demonstrated for the first time that rutin treatment was able to increase thyroid NIS expression and radioiodide uptake *in vivo*. This effect is of great interest, since the ability of the thyrocytes to uptake iodide is clinically useful, because therapy with radioiodine is used to treat thyroid diseases, such as hyperthyroidism and thyroid cancer [25–27]. Therefore, a compound able to stimulate thyroid iodide uptake could be useful as an adjuvant in the therapy with radioiodine, especially in the treatment of thyroid cancer [44–46]. Previous data have shown that the effects of flavonoids on thyroid radioiodide uptake are controversial. In FTC-133 cells transfected with hNIS, flavonoids differentially affected radioiodide uptake, depending on the flavonoid used, leading to increased, decreased or unaltered NIS [17]. Kang et al. (2011) [18] have shown that the flavonoids genistein and resveratrol were able to increase NIS expression in the thyroid cancer cell line FTC-133. Therefore, one could speculate that the treatment with rutin could be useful to improve radioiodine therapy. However, this hypothesis remains to be tested.

The increased NIS and TPO expression and activity found in rats treated with rutin might be due to increased sensitivity of the thyroid to TSH, since serum TSH levels remained unchanged, while mRNA and protein levels of TSH receptor were up-regulated in the thyroid of rats treated with rutin. However, the mechanism(s) responsible for the higher TSH receptor expression in the thyroid of rutin-treated animals remains to be elucidated. In addition, TSH bioactivity increase, relief of iodide inhibition through 6 Iodo-delta-lactone or iodohexadecanal [47] or increased serum TSH, albeit not statistically significant, could also play a role.

Since thyroid radioiodide uptake and expression of NIS, TPO and TSHR were significantly increased in rutin-treated rats, it would be expected that the serum levels of thyroid hormones would be increased in rats treated with rutin, however this was not observed. Surprisingly, serum thyroid hormones levels were significantly decreased by treatment with rutin. Our results corroborate previous studies showing that the consumption of plants rich in flavonoids, and treatment with isolated flavonoids are associated with decreased serum concentration of thyroid hormones *in vivo* [4,5,7,48,49]. Since rutin was able to potently inhibit TPO iodide-oxidation activity *in vitro*, it is likely that the same occurs *in vivo*. Therefore, the reduction of serum thyroid hormone concentration could be due, at least in part, to the *in vivo* TPO inhibition. As a result, intra glandular organified iodine (IX) content should be lower, and could explain the higher response to the TSH leading to increased NIS and TPO.

Rats treated with rutin were shown herein to have an increased BAT, hypothalamic and pituitary type 2 deiodinase activity. In 2007, Da-SILVA et al. [16] have demonstrated that some flavonols were able to increase D2 activity in rabdomyosarcoma (RMS-13) cell line. We could thus suggest that the increase in D2 activity of rutin-treated animals may be due to a direct effect of rutin, which is a flavonol, on type 2 iodothyronine deiodinase activity.

On the other hand, liver D1 activity was significantly decreased by treatment with rutin. The decrease of liver D1 activity could be important, at least in part, for the decrease in serum T3 concentration in rutin-treated animals, since liver D1 activity is important for peripheral T3 production [50], however it cannot explain the decreased T4 levels. Previously, our group has shown that rutin could inhibit thyroid D1 activity *in vitro* [15], suggesting a direct effect of rutin modulating hepatic D1 activity. It is also well-known that T3 has a stimulatory effect on hepatic D1 expression and activity [50]. Thus, reduced D1 activity in the liver of rutin-treated rats could be due to both reduced T3 levels found in these animals and/or to a direct inhibitory effect of rutin.

Despite the decreased serum thyroid hormone concentration in rutin-treated rats, TSH levels remained abnormally unchanged. Since rats treated with rutin showed an increase in hypothalamic and pituitary D2 activity, the increased intracellular availability of T3 in the hypothalamus and pituitary might have impaired the rise in TSH, since T3 locally produced by T4 deiodination seems to be of great importance for the negative feedback [50]. Therefore, the increment in the conversion of T4 to T3 in the hypothalamus and pituitary of rutin-treated rats due to the increased D2 activity could lead to greater suppression of the axis, thus preventing the rise in TSH levels despite the decreased serum thyroid hormone concentration.

## Conclusion

We have demonstrated herein that the short-term treatment with rutin *in vivo* was able to reduce serum thyroid hormone concentration, without affecting serum TSH levels. Thyroid peroxidase, the key enzyme in thyroid hormone biosynthesis, was strongly inhibited by rutin *in vitro*, and this is probably the cause for the decreased thyroid hormones found in rutin-treated rats. *In vivo* rutin treatment was also able to increase hypothalamic and pituitary type 2 iodothyronine deiodinase activity, which might be the cause for the abnormally unchanged serum TSH concentration. Despite the normal TSH levels, thyroid NIS and TPO expression was significantly increased *in vivo*, suggesting increased stimulation of the thyroid function, which might be due to increased TSH bioactivity or responsiveness, since thyroid TSH receptor expression was significantly increased in rutin-treated rats. It is important to underline that treatment with rutin for five days increased thyroid NIS protein and mRNA levels, besides increasing thyroid radioiodide uptake. Since radioiodine ablation is an important tool in the treatment of thyroid diseases, including thyroid cancer, we can speculate that rutin could be useful as an adjuvant for radioiodine therapy. However, more studies are necessary in order to better understand the mechanisms involved in the effect of rutin on thyroid iodide uptake.
