# Correction: Flavonoid Rutin Increases Thyroid Iodide Uptake in Rats

**DOI:** 10.1371/annotation/cc98ac47-e02a-400f-9213-05352b23a144

**Published:** 2014-01-06

**Authors:** Carlos Frederico Lima Gonçalves, Maria Carolina de Souza dos Santos, Maria Gloria Ginabreda, Rodrigo Soares Fortunato, Denise Pires de Carvalho, Andrea Claudia Freitas Ferreira

The names of multiple authors were incorrectly expressed in the Citation. The correct Citation is: Gonçalves CFL, Santos MCS, Ginabreda MG, Fortunato RS, Carvalho DP, et al. (2013) Flavonoid Rutin Increases Thyroid Iodide Uptake in Rats. PLoS ONE 8(9): e73908. doi:10.1371/journal.pone.0073908 

